# Efficacy, Safety and Steroid-sparing Effect of Topical Cyclosporine A 0.05% for Vernal Keratoconjunctivitis in Indian Children

**DOI:** 10.18502/jovr.v14i4.5439

**Published:** 2019-10-24

**Authors:** Arkendu Chatterjee, Sabyasachi Bandyopadhyay, Samir Kumar Bandyopadhyay

**Affiliations:** ^1^NRS Medical College & Hospital, Kolkata, India; ^2^R.G.Kar Medical College & Hospital, Kolkata, India

**Keywords:** Allergic Conjunctivitis, Intraocular Pressure, Topical Cyclosporine A, Topical Loteprednol Etabonate, Vernal Keratoconjunctivitis

## Abstract

**Purpose:**

To evaluate the efficacy, safety, and steroid-sparing effect of topical cyclosporine A (Cs A) 0.05% in patients with moderate to severe steroid dependent vernal keratoconjunctivitis (VKC).

**Methods:**

A prospective, comparative, placebo controlled study was carried out on 68 VKC patients, with 34 patients treated with topical Cs A 0.05% and the remaining 34 with topical carboxymethyl cellulose 0.5% (placebo). Both groups also received topical loteprednol etabonate 0.5%. Symptom (itching, photophobia, tearing, and discharge) score, sign (tarsal and limbal papillae, corneal involvement, and conjunctival hyperemia) score, and drug score (steroid drop usage/day/eye) were recorded at baseline and each follow-up visit. The intraocular pressure (IOP) measurement and evaluation of any ocular side effects were carried out.

**Results:**

Significant reduction in symptom score and sign score was seen in both groups. Cs A group significantly showed more reduction in symptom (*P* < 0.0001 in all follow-up visits) and sign (*P* < 0.0001 in all follow-up visits) scores compared to the placebo group. At day 7, mean steroid usage reduced from 4 to 3.44 ± 0.5 and 3.79 ± 0.4 in Cs A and placebo groups, respectively (*P* < 0.0001). Steroid drops completely stopped in 21 patients at day 60 in the Cs A group compared to none in the placebo group. No significant rise in IOP or any side effects were noted in either group.

**Conclusion:**

Topical Cs A 0.05% is effective and safe in patients with moderate to severe VKC with good steroid-sparing effect.

##  INTRODUCTION

Vernal keratoconjunctivitis (VKC) is a seasonal bilateral chronic allergic inflammatory disease of the ocular surfaces, mainly occurring in children and adolescents living in dry and temperate regions.^[1,2]^ Itching, photophobia, tearing, and mucoid discharge may occur in patients with VKC.^[3]^ Superior tarsal and limbal papillae, conjunctival hyperemia, and corneal involvement in the form of punctate epithelial keratitis, epithelial macroerosions, shield ulcers, plaque formation, and corneal neovascularization are also observed.^[4]^ VKC can be described as IgE and T-cell mediated ocular allergic reaction with varied etiological factors comprising environmental allergens, climate, and genetic predisposition.^[5]^ Mast cells, eosinophils, and their mediators play a major role in the clinical manifestation of the disease.^[5]^

Topical mast cell stabilizers and antihistaminic drugs (sodium cromoglycate 2% and olapatadine 0.1%) are the first line of drugs used in mild cases, but in moderate to severe cases, additional topical steroids such as prednisolone and dexamethasone or other immunomodulators such as cyclosporine A (Cs A) and tacrolimus may be needed.^[6,7]^ However, topical prednisolone and dexamethasone are associated with ocular adverse reactions, including increase in intraocular pressure (IOP);^[8]^ therefore, 0.5% topical loteprednol etabonate (LE), with a relatively milder effect on IOP, has been used in the treatment of VKC with good efficacy and less side effects.^[9,10]^ LE is a novel corticosteroid manufactured via retrometabolic design, which is rapidly metabolized by tissue esterases to D1 cortienic acid etabonate and eventually to D1 cortienic acid, thereby reducing any potential side effects. Despite its relatively safer applications, LE should still be used with caution, since a significant rise in IOP due to LE administration has been reported in allergic conjunctivitis as compared to that with placebo or olopatadine.^[11]^ Cs A is an immunosuppressive molecule, which reduces ocular inflammation by inhibiting Th2 lymphocyte proliferation, interleukin-2 production, and histamine release from mast cells and basophils.^[12,13]^ Several studies have been performed with topical Cs A 0.05% in moderate to severe steroid dependent (LE, fluorometholone acetate, or prednisolone acetate) VKC patients with good efficacy of treatment and reduction in doses of topical steroids.^[4,14,15]^

In the present study, we aimed to evaluate the safety, efficacy, and possible steroid-sparing effect of topical Cs A 0.05% in an aqueous solution compared to placebo in Indian children with VKC concurrently treated with topical LE 0.5%.

##  METHODS

The present study is a prospective comparative, placebo-controlled study of 68 patients (aged 5–15 years) with moderate to severe VKC, who visited a tertiary care hospital in eastern India from January 1, 2017 to June 30, 2017. Patients were randomly allocated either into Cs A group (34 patients treated with topical Cs A 0.05% plus topical LE 0.5%) or placebo group (34 patients treated with carboxymethyl cellulose 0.5% and topical LE 0.5%) according to a computer generated predetermined randomization list. VKC was diagnosed based on the presence of itching, tearing, mucoid discharge, photophobia, tarsal and limbal papillae, corneal involvement, and conjunctival hyperemia. Patients using systemic steroids or any immunosuppressive drugs or non-steroidal anti-inflammatory medications, or having associated corneal diseases, uveitis, glaucoma, and optic atrophy were excluded from the study. All the participating patients were diagnosed with active disease at the time of enrolment. Any prior topical medication for VKC was stopped for three days and only physical measures were advised during that period. Thereafter, topical eye drops were instituted according to the protocol. Both eyes of each patient were examined. The study was carried out in accordance with the ethical principles outlined in the Declaration of Helsinki and informed parental consent was obtained. The protocol was approved by the Institutional ethics committee.

Patients in the Cs A group were treated with aqueous ophthalmic solution of topical Cs A 0.05% (Hydroeyes 0.05% w/vⓇ Lupin Ltd., Mumbai, India) in either eye with one drop four times/day, and those in the placebo group were treated with topical carboxymethyl cellulose 0.5% (Refresh tearsⓇ Allergan Inc., Irvine, CA, USA) in either eye with one drop four times/day. Both groups received one drop of topical LE (0.5%) (LotepredⓇ Sun Pharma (Avesta), Mumbai, India) four times daily initially, and the dose then tapered off from the first follow-up at day 7 according to the clinical response. Steroid dosage reduction was approved if both the sign and symptom scores were ≤ 4 and steroid was discontinued if both the sign and symptom scores were ≤ 1. Follow-up check-ups were carried out on days 7, 14, 30, 60, and 90.

At baseline (day 0), detailed demographic information, clinical history, and specific symptoms of the patients were obtained. A complete ophthalmological examination including visual acuity determination by Snellen chart, anterior segment evaluation by slit lamp biomicroscopy, IOP measurement by Goldmann Applanation Tonometer, and indirect ophthalmoscopy were performed. The same examination procedures were repeated in each follow-up visit. Scores ranging 0 to 3 (according to severity) were assigned to each symptom (itching, tearing, photophobia, and discharge) and sign (tarsal papillae, corneal involvement, limbal papillae, and conjunctival hyperemia), and total symptom and sign scores were calculated at each visit, similar to that of Ozlem et al [Table 1].^[4]^ Statistical calculations were based on these evaluated scores.

**Table 1:** The scoring method of symptoms and signs of vernal keratoconjunctivitis


**Variable**	**Score**			
	**0**	**1**	**2**	**3**
**Symptom**				
Itching	None	Occasional	Frequent	Constant
Tearing	Normal	Mild	Moderate	Severe
Photophobia	None	Mild	Moderate	Severe
Discharge	None	Small	Moderate	Profuse
**Sign**				
Conjunctival hyperemia	None	Mild	Moderate	Severe
Tarsal papillae	None	< 1 mm	1–3 mm	> 3 mm
Limbal papillae	None	< 2 mm or < 90°	2–4 mm or 90°–180°	> 4 mm or > 180°
Corneal involvement	Normal cornea	Fine SPEK*	Coarse SPEK/macro erosion	Shield ulcer/pannus
	
	
*SPEK, superficial punctate epithelial keratitis				

###  Statistical Analysis

The collected data were incorporated into Microsoft Excel 2007 (Microsoft Corporation, Redmond, WA, USA) worksheet and analyzed using SPSS version 15.0 (SPSS Inc., Chicago, IL, USA). Simple correlations and linear regressions were performed between both eyes for validation of data obtained from either eye. To compare the differences between and within the groups, Friedman test, ANOVA with Bonferroni's post-hoc test, Wilcoxon and Kruskal–Wallis test for categorical variables, and repeated measurement ANOVA for continuous variables were performed. A *P*-value < 0.05 was considered statistically significant.

##  RESULTS 

**Figure 1:**
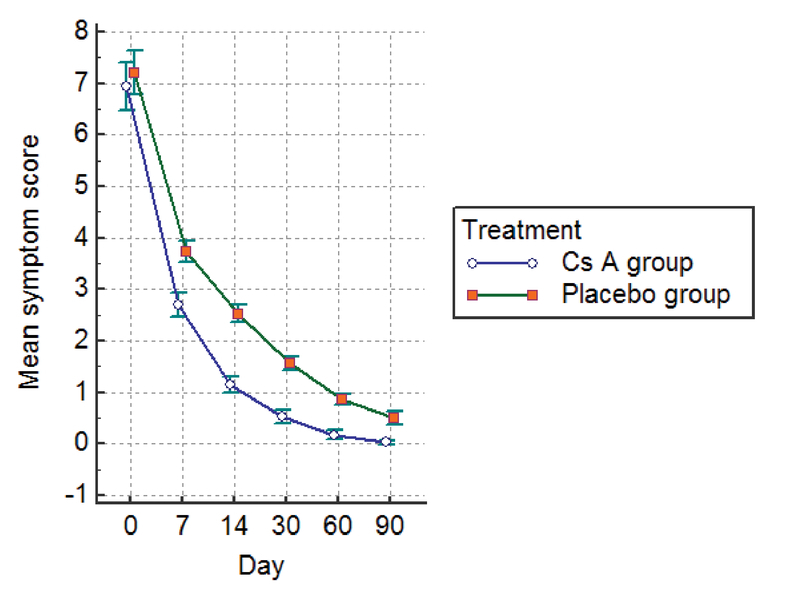
Mean symptom scores at baseline and during follow-up visits.

The patients in Cs A group comprised 20 males and 14 females with mean age of 10.23 ± 2.7 years. The placebo group had 21 males and 13 females with mean age of 10.35 ± 3.26 years. At day 0 (baseline), there were no significant differences between the mean symptom scores (6.94 ± 1.9 and 7.2 ± 1.77 in Cs A and placebo groups, respectively; *P* = 0.4037) and mean sign scores (7.14 ± 2.13 and 6.88 ± 1.8 in Cs A and placebo groups, respectively; *P *= 0.4363) in either group. Mean symptom score in Cs A group at day 7 reduced to 2.7 ± 0.96 with further reduction observed upon subsequent visits (*P* < 0.0001 in all cases) [Figure 1]. Mean symptom score in placebo group also showed reduction at day 7 (3.73 ± 0.89), which further decreased in follow-up visits (*P* < 0.0001 in all cases). A comparison between the two groups indicated that Cs A group showed higher symptom score reduction over the placebo group in all follow-up assessments (*P* < 0.0001 in all cases).

**Figure 2:**
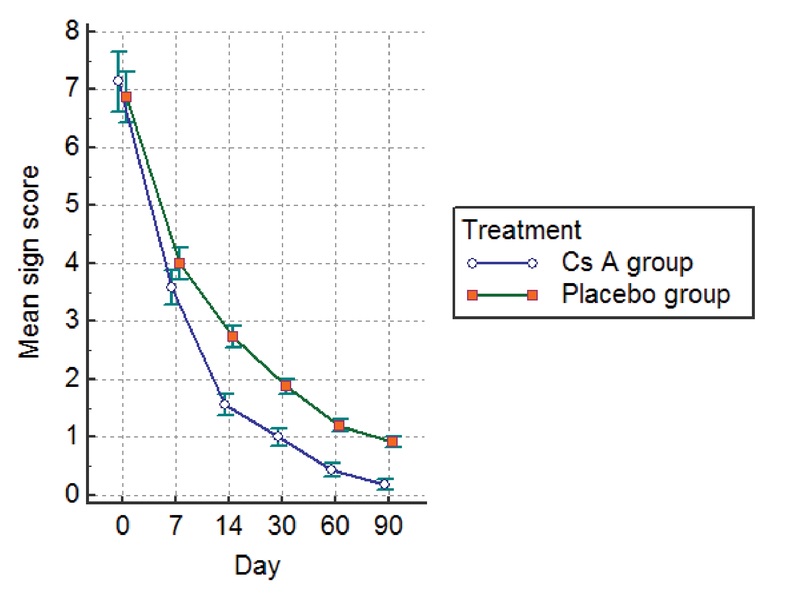
Mean sign scores at baseline and during follow-up visits.

Mean sign score in Cs A group decreased to 3.58 ± 1.19 at day 7 with continued reduction observed upon subsequent visits (*P* < 0.0001 in all cases) [Figure 2]. Similarly, in the placebo group, mean sign score showed reduction at day 7 (4.00 ± 1.11), which further decreased in follow-up visits (*P* < 0.0001 in all cases). Cs A group showed more reduction in sign score over placebo group from day 7 (*P *= 0.04), which was more evident from day 14 (*P* < 0.0001 in all cases).

**Figure 3:**
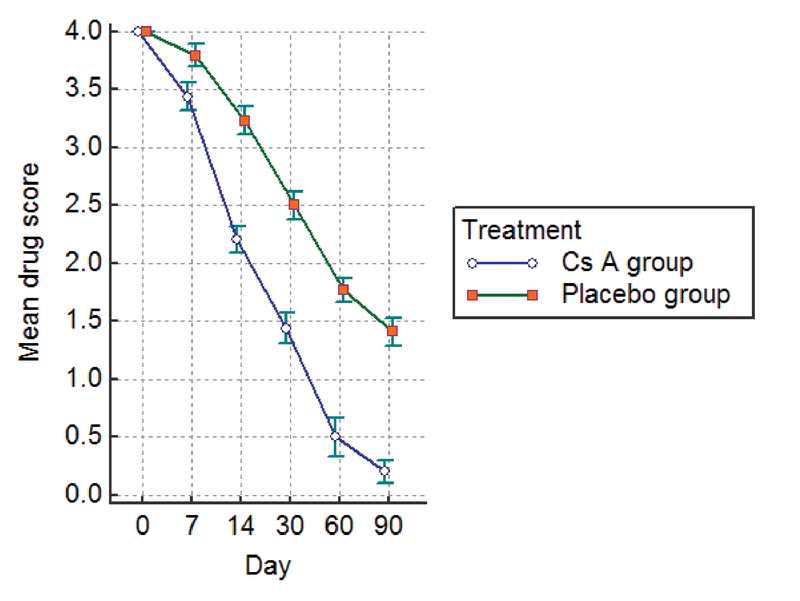
Mean steroid drop usage per day per eye (drug score) at baseline and during follow-up visits.

Initial steroid drop usage score was four drops per eye per day in both groups. In follow-up visits, there was a higher reduction in steroid drop usage in Cs A group (3.44 ± 0.5) at day 7 than in the placebo group (3.79 ± 0.4; *P* < 0.0001) [Figure 3]. This trend persisted in all the subsequent visits with complete termination of steroid drop use in 21 patients in the Cs A group as compared to none in the placebo group at day 60.

Mean IOP at baseline in Cs A group was 16.7 ± 2.3 mm Hg in the right eye and 16.58 ± 2.28 mm Hg in the left eye, which did not show any significant increase at day 30 (16.23 ± 2.24 mm Hg in the right eye, *P *= 0.1988 and 16.52 ± 1.86 mm of Hg in the left eye, *P* = 0.8723). In the placebo group, the mean IOP at baseline was 17.11 ± 2.51 mm Hg in the right eye and 16.82 ± 2.61 mm Hg in the left eye, which also did not show any significant increase at day 30 (17.52 ± 1.97 mm Hg in the right eye, *P* = 0.2558 and 17 ± 1.98 mm of Hg in the left eye, *P* = 0.6809). No side effects were reported in either group during the follow-up visits.

##  DISCUSSION

The treatment of VKC is aimed at controlling the symptoms and prevention of complications. In mild cases, topical mast cell stabilizers and antihistaminics may be sufficient, but moderate to severe cases require topical steroids for adequate control.^[6,7]^ However, long-term topical steroid use, particularly that of dexamethasone phosphate and prednisolone acetate, is restricted due to the known side effects, such as increased IOP usually after the second week of therapy.^[8]^ Newer synthetic corticosteroids, such as LE (0.5%), which show relatively lower propensity for increase in IOP and are more effective in VKC, also show some side effects.^[9,11]^ In this context, reduction in dosage of topical LE is essential after one week of therapy in steroid-dependent and steroid-resistant VKC cases. Topical Cs A has been found to be a suitable alternative, either upon single use or as an adjunct to steroid therapy aimed at reducing the dosage of steroids.^[4,16]^ Earlier studies with high concentrations of up to 2% Cs A have shown effectiveness with VKC, although they were less tolerated due to stinging and burning sensations probably attributed to the use of maize or olive oils as vehicles.^[17,18,19]^ Newer aqueous formulations of Cs A in lower concentrations (0.1% and 0.05%) were found to be safe, effective, and well tolerated in VKC patients.^[4,16]^ These formulations have been observed to be good steroid sparing agents in steroid responsive VKC patients, but their optimal effect in reducing the signs and symptoms was achieved only after two weeks of therapy.^[4,16][17,18,19]^ Hence, we used the combination of 0.5% LE with 0.05% Cs A for a prompt initial response and also to attempt to lower the frequency of steroid drop usage along with Cs A after the first week of therapy depending upon the clinical response.

We observed a significant improvement in symptoms and signs of VKC in both groups; however, the Cs A group showed more pronounced improvement as compared to the placebo group, which was similar to the observations made by Baiza-Duran et al and Ozlem et al.^[4,16]^ Both groups showed no serious side effects, including no increase in IOP, which was corroborative with Daniell et al, Baiza-Duran et al, and Ozlem et al.^[4,14,16]^

In this study, we observed that the aqueous formulation of Cs A 0.05% is more effective in reducing steroid drops in VKC as compared to placebo since the first week of therapy and complete termination of steroid therapy was noted in 21 out of 34 patients in the Cs A group as compared to no patients discontinuing steroid dosage in the placebo group at day 60. In a recent study with 30 VKC patients, Ozlem et al observed that Cs A 0.05% can help in reducing corticosteroid usage and is a safe and effective alternative for the treatment of resistant VKC.^[4]^ Baiza-Duran et al compared 0.1% and 0.05% aqueous formulation of Cs A in 112 Mexican children with VKC and found that both concentrations were safe, effective, and equally well-tolerated.^[16]^ Keklikci et al studied the efficacy of topical Cs A 0.05% in patients with severe VKC and noted significant clinical improvement as well as decreased density of inflammatory cells in conjunctival impression cytology specimens.^[20]^ Akpek et al performed a randomized trial with topical Cs A 0.05% in topical steroid resistant atopic keratoconjunctivitis. They found that topical Cs A 0.05% was safe and effective in alleviating signs and symptoms of severe AKC that was refractory to topical steroid treatment.^[21]^

In another study, De Smedt et al examined patients with VKC in Rwanda, Central Africa and found no significant differences in terms of efficiency between 2% topical Cs A and 0.1% dexamethasone in the management of acute VKC.^[22]^ However, Cs A drops caused more stinging in patients than the oil placebo and dexamethasone.

In a retrospective review on the use of 0.05% topical Cs A for pediatric allergic conjunctivitis in Chinese patients in Hong Kong, Wu MM et al found it to be significantly effective and safe in reducing ocular symptom and sign scores three months after use.^[23]^

Keklikci et al in their placebo-controlled, randomized prospective study of 62 patients with VKC observed that 0.05% topical Cs A eye drops were safe and effective in the treatment of patients with VKC.^[24]^ However, Daniell et al observed that 0.05% topical Cs A had no benefit over placebo as a steroid sparing agent in 18 AKC and 22 VKC patients.^[14]^ They opined that the dosage of Cs A might have been insufficient or use of steroid might have masked the benefits of Cs A.

The limitation of our study is that we did not analyze recurring cases after cessation of therapy. In our study period, we did not come across any recurrence during treatment. However, the study would have been strengthened had we included the evaluation of any recurrences, which is definitely an important aspect to be considered for a future continuation study.

In conclusion, topical application of 0.05% Cs A in an aqueous preparation as one drop four times a day along with topical LE 0.5% is an effective and safe treatment module for patients with moderate to severe VKC, with good steroid sparing effect. Further completely randomized, double-blind, placebo-controlled studies with larger samples and longer follow-up periods are required to determine the optimal duration of treatment and likelihood of recurrence after cessation of therapy with Cs A.

## Financial Support and Sponsorship

Nil.

## Conflicts of Interest

There are no conflicts of interest.

## References

[1] Leonardi A. (2002). Vernal keratoconjunctivitis: pathogenesis and treatment. *Progress in Retinal and Eye Research*.

[2] Leonardi A., Secchi A. G. (2003). Vernal keratoconjunctivitis. *International Ophthalmology Clinics*.

[3] Vichyanond P., Pacharn P., Pleyer U., Leonardi A. (2014). Vernal keratoconjunctivitis: A severe allergic eye disease with remodeling changes. *Pediatric Allergy and Immunology*.

[4] Yücel O., Ulus N. (2016). Efficacy and safety of topical cyclosporine A 0.05% in vernal keratoconjunctivitis. *Singapore Medical Journal*.

[5] Leonardi A. (2013). *Management of Vernal keratoconjunctivitis. Ophthalmol Ther*.

[6] Allansmith M. R., Ross R. N. (1986). Ocular allergy and mast cell stabilizers. *Survey of Ophthalmology*.

[7] Gokhale N. (2016). Systematic approach to managing vernal keratoconjunctivitis in clinical practice: Severity grading system and a treatment algorithm. *Indian Journal of Ophthalmology*.

[8] Mcghee C. N., Dean S., Danesh-Meyer H. (2002). Locally Administered Ocular Corticosteroids. *Drug Safety*.

[9] Öner V., Türkcü F. M., Taş M., Alakuş M. F., İşcan Y. (2012). Topical loteprednol etabonate 0.5 % for treatment of vernal keratoconjunctivitis: efficacy and safety. *Japanese Journal of Ophthalmology*.

[10] Eliaçik M., Erdoğan F., Bayramlar H., Karaman S., Gülkilik G. (2014). Comparison of efficacy and safety of Loteprednol etabonate 0.5% versus fluorometholone acetat 0.1% for the treatment of vernal keratoconjunctivitis in pediatric subjects. *Nobel Medicus*.

[11] Wu L., Chen X., Lou H., Cheng J., Wei R. (2015). Loteprednol etabonate in the treatment of allergic conjunctivitis: a meta-analysis. *Current Medical Research and Opinion*.

[12] Fukushima A., Yamaguchi T., Ishida W., Fukata K., Liu F., Ueno H. (2006). Cyclosporin A inhibits eosinophilic infiltration into the conjunctiva mediated by type IV allergic reactions. *Clinical & Experimental Ophthalmology*.

[13] Saari K. M. Updates in the treatment of ocular allergies. *Journal of Asthma and Allergy*.

[14] Daniell M. (2006). Randomised controlled trial of topical ciclosporin A in steroid dependent allergic conjunctivitis. *British Journal of Ophthalmology*.

[15] Tatlipinar S., Akpek E. K. (2005). Topical ciclosporin in the treatment of ocular surface disorders. *British Journal of Ophthalmology*.

[16] Baiza-Duran L. M., González-Villegas A. C., Contreras-Rubio Y., Juarez-Echenique J. C., Vizzuett-Lopez I. V. (2010). Safety and Efficacy of Topical 0.1% And 0.05% Cyclosporine A in an Aqueous Solution in Steroid-Dependent Vernal Keratoconjunctivitis in a Population of Mexican Children. *Journal of Clinical & Experimental Ophthalmology*.

[17] Hingorani M., Moodaley L., Calder V. L., Buckley R. J., Lightman S. (1998). A randomized, placebo-controlled trial of topical cyclosporin A in steroid-dependent atopic keratoconjunctivitis. *Ophthalmology*.

[18] Pucci N., Novembre E., Cianferoni A., Lombardi E., Bernardini R., Caputo R., Campa L., Vierucci A. (2002). Efficacy and safety of cyclosporine eyedrops in vernal keratoconjunctivitis. *Annals of Allergy, Asthma & Immunology*.

[19] Spadavecchia L., Fanelli P., Tesse R., Brunetti L., Cardinale F., Bellizzi M., Rizzo G., Procoli U., Bellizzi G., Armenio L. (2006). Efficacy of 1.25% and 1% topical cyclosporine in the treatment of severe vernal keratoconjunctivitis in childhood. *Pediatric Allergy and Immunology*.

[20] Keklikci U., Soker S. I., Sakalar Y. B., Unlu K., Ozekinci S., Tunik S. (2008). Efficacy of topical cyclosporin A 0.05% in conjunctival impression cytology specimens and clinical findings of severe vernal keratoconjunctivitis in children. *Japanese Journal of Ophthalmology*.

[21] Akpek E. K., Dart J. K., Watson S., Christen W., Dursun D., Yoo S., O'Brien T. P., Schein O. D., Gottsch J. D. (2004). A randomized trial of topical cyclosporine 0.05% in topical steroid-resistant atopic keratoconjunctivitis. *Ophthalmology*.

[22] De Smedt S., Nkurikiye J., Fonteyne Y., Tuft S., De Bacquer D., Gilbert C., Kestelyn P. (2012). Topical ciclosporin in the treatment of vernal keratoconjunctivitis in Rwanda, Central Africa: a prospective, randomised, double-masked, controlled clinical trial. *British Journal of Ophthalmology*.

[23] Wu Macy M. S., Yau Gordon S. K., Lee Jacky W. Y., Wong Amy L., Tam Victor T. Y., Yuen Can Y. F. (2014). Retrospective Review on the Use of Topical Cyclosporin A 0.05% for Paediatric Allergic Conjunctivitis in Hong Kong Chinese. *The Scientific World Journal*.

[24] Keklikci U., Dursun B., Cingu A. K. (2014). Topical cyclosporine a 0.05% eyedrops in the treatment of vernal keratoconjunctivitis—randomized placebo-controlled trial. *Advances in Clinical and Experimental Medicine*.

